# Time Series Transcriptome Analysis in *Medicago truncatula* Shoot and Root Tissue During Early Nodulation

**DOI:** 10.3389/fpls.2022.861639

**Published:** 2022-04-07

**Authors:** Yueyao Gao, Bradley Selee, Elise L. Schnabel, William L. Poehlman, Suchitra A. Chavan, Julia A. Frugoli, Frank Alex Feltus

**Affiliations:** ^1^Department of Genetics and Biochemistry, Clemson University, Clemson, SC, United States; ^2^Department of Electrical and Computer Engineering, Clemson University, Clemson, SC, United States; ^3^Sage Bionetworks, Seattle, WA, United States; ^4^Biomedical Data Science and Informatics Program, Clemson University, Clemson, SC, United States; ^5^Clemson Center for Human Genetics, Greenwood, SC, United States

**Keywords:** differential gene expression, nodulation, rhizobia, *Medicago truncatula*, transcriptional dynamic, time series

## Abstract

In response to colonization by rhizobia bacteria, legumes are able to form nitrogen-fixing nodules in their roots, allowing the plants to grow efficiently in nitrogen-depleted environments. Legumes utilize a complex, long-distance signaling pathway to regulate nodulation that involves signals in both roots and shoots. We measured the transcriptional response to treatment with rhizobia in both the shoots and roots of *Medicago truncatula* over a 72-h time course. To detect temporal shifts in gene expression, we developed GeneShift, a novel computational statistics and machine learning workflow that addresses the time series replicate the averaging issue for detecting gene expression pattern shifts under different conditions. We identified both known and novel genes that are regulated dynamically in both tissues during early nodulation including leginsulin, defensins, root transporters, nodulin-related, and circadian clock genes. We validated over 70% of the expression patterns that GeneShift discovered using an independent *M. truncatula* RNA-Seq study. GeneShift facilitated the discovery of condition-specific temporally differentially expressed genes in the symbiotic nodulation biological system. In principle, GeneShift should work for time-series gene expression profiling studies from other systems.

## 1. Introduction

As most biological processes are dynamic, time-series transcriptome profiling experiments play a pivotal role in understanding and modeling these processes. However, understanding the dynamics of a transcriptional response is still a major challenge. Many time-series transcriptomics experiments apply differentially expressed gene (DEG) detection software packages that only detect the gene expression change between conditions at each time point. Single time point DEG detection does not emphasize the sequential nature of time-series data (Larrainzar et al., 2015; Schiessl et al., 2019). In contrast, full time-series profile DEGs that exhibit differential expression patterns across the time interval under different conditions will help one understand more about cellular response to environmental signals at the transcriptomic level.

Some time-series specific approaches deployed for transcriptomic data do not consider the dynamics of gene expression under two conditions (Jung et al., 2017; Bacher et al., 2018; McDowell et al., 2018). Replicates also play a key role in time-series experiments, but simply averaging the expressions at each time point can be very misleading (Celeux et al., 2005; Nguyen et al., 2010). It is a challenge for time-series analysis software to treat time-series replicates as multiple individual gene quantifications. To address these issues, we developed a computational workflow called GeneShift, which focuses on time-series pattern detection without averaging replicates. GeneShift is able to detect time-series DEGs between biological conditions which we demonstrate using a *Medicago truncatula* experimental system.

The root nodulation process consists of a series of interactions between legumes and compatible rhizobial symbiotic bacteria, including nodule formation, partner selection, suppression of plant defense responses, and autoregulation of nodulation (Ferguson et al., 2010; Hayashi et al., 2014; Sprent et al., 2017). Understanding the transcriptional reprogramming associated with nodulation is a powerful approach to decipher the genetic control of nodulation and, thus, to engineer the beneficial nitrogen-fixing symbiosis into non-leguminous crops. Over the past two decades, many transcriptome profiling studies have been conducted to identify the responsible genes and cellular processes involved in nodulation (Fedorova et al., 2002; El Yahyaoui et al., 2004; Lee et al., 2004; Benedito et al., 2008; Høgslund et al., 2009; Libault et al., 2010; Maunoury et al., 2010; Breakspear et al., 2014; Roux et al., 2014; Larrainzar et al., 2015; Jardinaud et al., 2016; Schiessl et al., 2019). Although previous transcriptomic studies have substantially transformed our understanding of nitrogen-fixing symbiosis, most of them have only profiled the transcriptional changes of the root at one or two time points during the nodulation process. Thus, many genes involved in the nodulation may not have been captured in these analyses due to the insufficient temporal resolution or missing corresponding control conditions.

**Figure 1:**
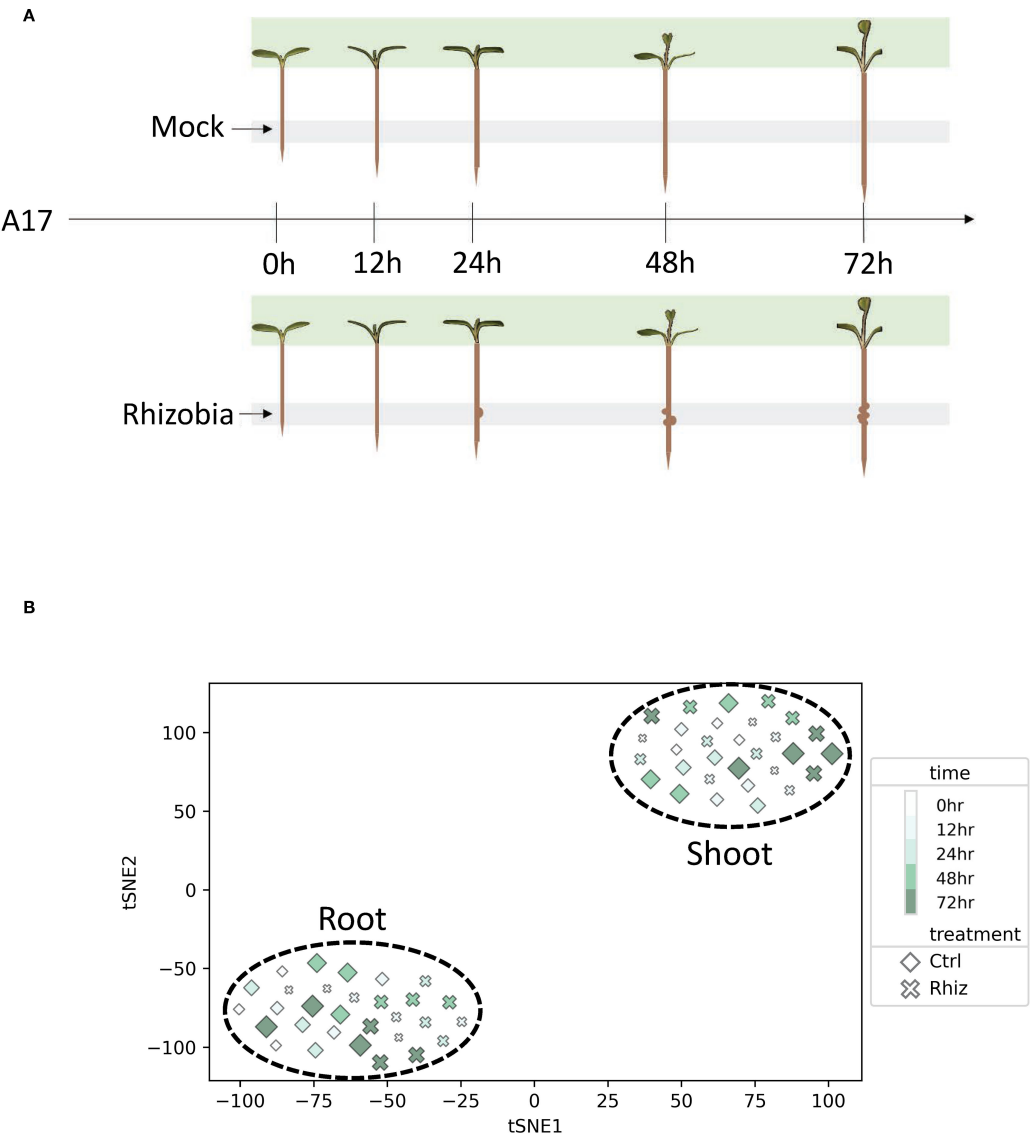
Root and shoot displayed transcriptional differences in control and rhizobia samples. **(A)** Experimental design for the *Medicago truncatula* transcriptomics experiment. Gray and green shades represent harvest tissue locations for RNA-seq library construction. **(B)** T-distributed stochastic neighbor embedding (tSNE) reveals the transcriptional difference between rhizobia and control samples in root vs. shoot.

**Figure 2:**
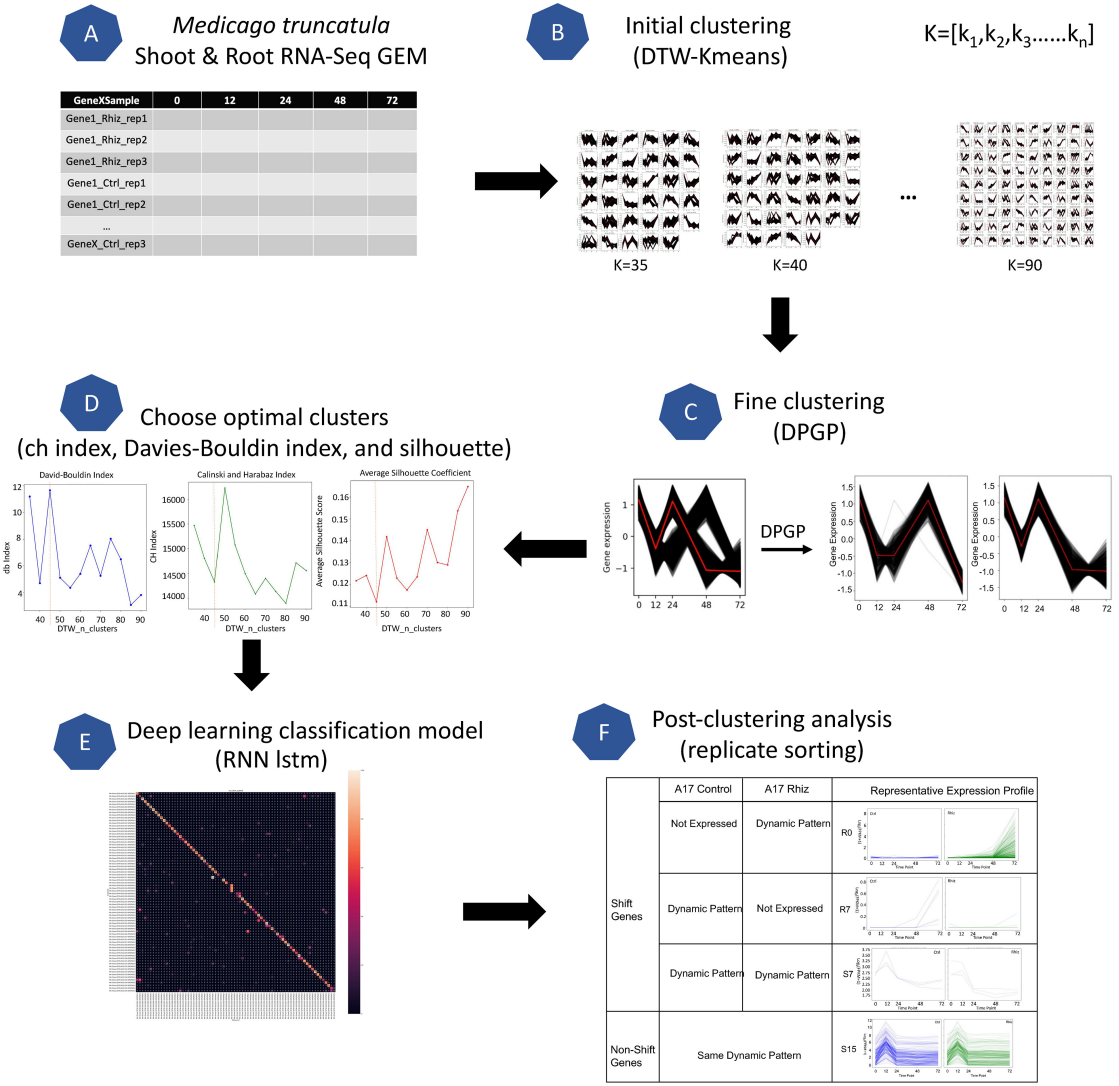
Overview of the GeneShift workflow. Major steps of the workflow are shown in steps **(A–F)**. Starting with RNA-Seq data from a biological sample, GeneShift will detect gene expression pattern changes over time between two conditions. Refer to Materials and Methods for details.

**Figure 3:**
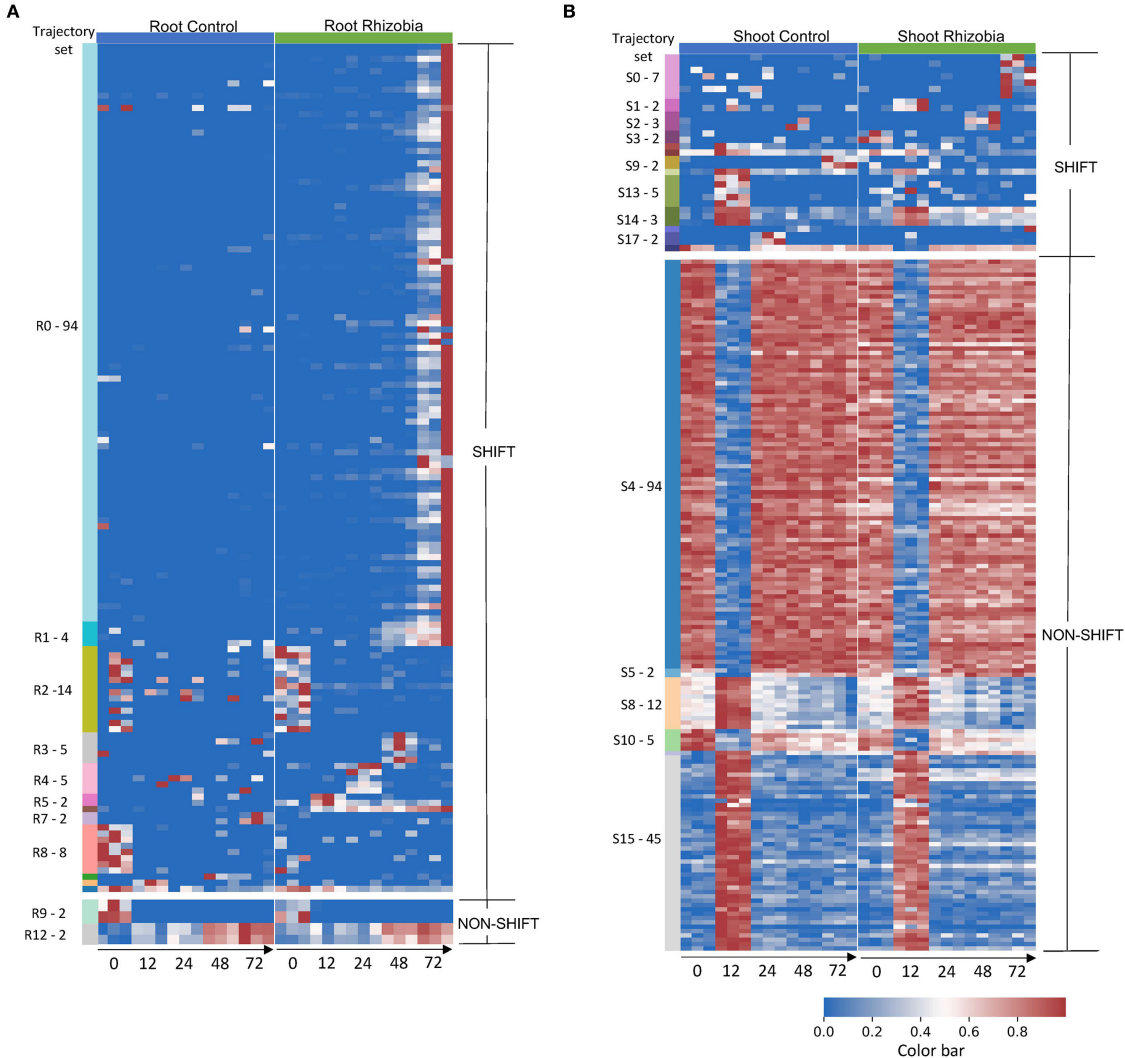
Gene expression profile in root and shoot of control and rhizobia-infected Medicago plants over 72 h. **(A)** Heatmap of 142 root genes and **(B)**. Heatmap of 190 shoot genes that GeneShift identified. Data are presented in the heatmap using log2 (x+1) transformed FPKM expression values. Each row represents a gene, and each column represents the expression profile for a single biological sample. The color reference to the left of each heatmap is representing GeneShift trajectory groups (R = root, S = shoot) and the number of genes in that trajectory set. The trajectory sets contain only one gene were not annotated. The numbers below the heatmap represent hours post inoculation (hpi). The color bar represents the relative gene expression in a row.

In this study, we applied our GeneShift workflow to study the nitrogen-fixing symbiosis between *M. truncatula* and rhizobium *Sinorizobium medicae*. We generated RNA-Seq datasets across five time points in both control and rhizobia-treated roots and shoots (Figure 1). Our computational approach detected both known and unknown genes with different expression patterns under different conditions (Figures 2, 3). Finally, we employed previously published RNA-Seq data to validate the patterns we observed in *M. truncatula* root sections (Schiessl et al., 2019).

## 2. Results

For this study, we designed and implemented a workflow whereby time-series gene expression data can be used to identify full time interval DEGs. We chose to apply this workflow to analyze the rhizobia response in *M. truncatula* roots and shoots as shown in Figure 1A. We generated RNA-Seq data to measure transcriptional profiles of *M. truncatula* plants treated with either *S. medicae* or mock inoculation as the control for three biological replicates at each time point [0, 12, 24, 48, 72 h post inoculation (hpi)]. Defined root segments were harvested for the root samples, and the entire above-ground shoot was harvested for the shoot samples.

We constructed a gene expression matrix (GEM) comprised of 50,984 *M. truncatula* genes measured in triplicate for two treatments and five time points for both root and shoot as depicted by Supplementary Figure 1. T-distributed stochastic neighbor embedding (tSNE) was used to visualize the global similarities between the samples (Figure 1B). The samples were separated into two distinct and non-overlapping groups based on the shoot and root tissues. tSNE also showed some separation between control and rhizobia root samples compared to shoot samples.

To identify *M. truncatula* genes that were differentially regulated in treated root and shoot tissues compared to corresponding controls, we developed GeneShift, a workflow to detect pattern changes in a time-series experiment (Figure 2). In our use case, GeneShift first clustered time course expression patterns for each replicate using two clustering algorithms, DTW-KMeans (Cuturi and Blondel, 2017) and DP_GP (McDowell et al., 2018), for control and rhizobia-treated samples based on discrete expression trajectories as depicted in Figures 2A–D. Next, GeneShift applied a recurrent neural network long short-term memory (RNN-lstm) artificial intelligence (AI) learning model to verify the clustering accuracy of GeneShift (Figure 2E). By comparing gene trajectories under two conditions, GeneShift obtained the transition status for all genes, which were used to identify trajectory sets and individual DEGs (Figure 2F). More details of the workflow are reported in the Materials and Methods. We note that GeneShift should work for any RNA-Seq time-series experiment.

Table 1 provides a global view of transcriptional dynamics response to rhizobia in *M. truncatula* yielded by GeneShift. GeneShift identified 142 root genes and 190 shoot genes using three out of three replicate sorting (refer to Materials and Methods; Supplementary Tables 1–4). We then compared rhizobia and control expression trajectories in both tissues. This analysis captured 138 root genes and 31 shoot genes that had shifted expression patterns between the rhizobia treated and the control samples (Table 1). The shifted genes in both tissues were grouped into three different categories: ***A***. *transitions from control not expressed to a rhizobia dynamic pattern*, ***B***. *transitions from a control dynamic pattern to rhizobia not expressed, and* ***C***. *transitions from a control dynamic pattern to a rhizobia dynamic pattern*. Ninety percent of the root-shifted genes were upregulated under rhizobia treatment. By contrast, only 12 root genes were downregulated under rhizobia treatment including plant defensin MtDef4.3 (Medtr8g070780). The root shifted gene set and shoot shifted gene set were very tissue-specific and no shifted genes were shared between sets. GeneShift also captured four root genes and 159 shoot genes that maintained the same temporal expression pattern within 72 hpi with or without rhizobia treatment. Fewer expression changes in the shoot are expected because unlike the root, the shoot does not have a tissue reorganization response to rhizobia.

**Table 1:** *Medicago truncatula* GeneShift results summary for 3/3 replicates.

**Number of Genes**	**Root**	**Shoot**
All *M. truncatula* Genes	50,894	50,894
Not Expressed Control Genes Across All Time Points in 1+ Replicates	25,507	23,969
Not Expressed Rhizobia-treated Genes Across All Time Points in 1+ Replicates	23,666	23,815
Genes with 3 Consistent Replicates in both Conditions	142	190
Shift Genes between Control and Rhizobia Conditions	138	31
Genes Shift from Control Not Expressed to Rhizobia Dynamic Pattern	125	14
Genes Shift from Control Dynamic Pattern to Rhizobia Not Expressed	12	11
Genes Shift from Control Dynamic Pattern to Rhizobia Dynamic Pattern	1	6
Non-shift Genes between Control and Rhizobia Conditions	4	159

*The underlines represent the expression pattern of genes under specific conditions*.

**Figure 4:**
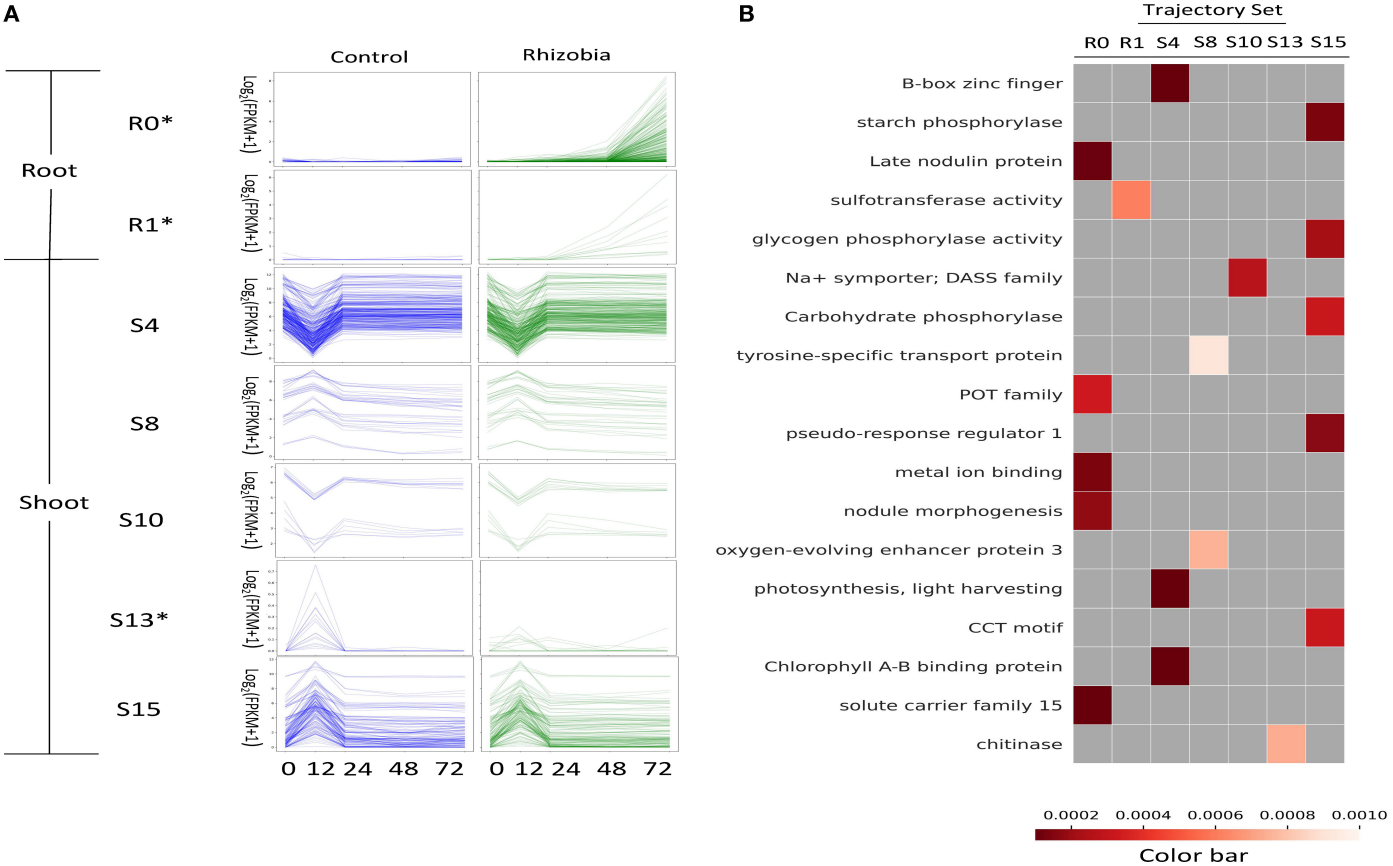
Functional enrichment of GeneShift output. **(A)** Expression profiles of several enriched GeneShift trajectory sets corresponding to Figure 3. Control uninoculated is blue, and inoculated with rhizobia is green. The unit of the y-axis is log2 (FPKM+1), and the unit of the x-axis is time (h) after rhizobia inoculation. Each thin line represents one replicate. The asterisk next to the trajectory set name indicates the pattern shifting between two conditions. **(B)** Gene Ontology, KEGG, fragments per kilobase of gene per million read pairs (FPKM) enrichment analysis of genes assigned to each trajectory set with Benjamini and Hochberg corrected *p* < 0.001. Each column of the heatmap indicates one trajectory set; each row presents one enriched term.

To examine the variation in gene expression over 72 hpi, we visualized the expression patterns of the 142 root genes and 190 shoot genes that GeneShift identified. There were 14 trajectory sets in the root (R0–R13) and 19 trajectory sets in the shoot (S0–S18), with two predominant patterns (shift and non-shift) (Figure 3). Genes in the largest root trajectory set (R0 with 94 genes in Figure 4A) were predominately upregulated at 72 hpi in rhizobia-inoculated roots, including the leginsulin related Legin47 (Medtr0112s0040) and Legin31 (Medtr8g022430). This trajectory set also includes genes for the vacuolar iron transporter like VTL4 (Medtr4g094325), early nodulin protein ENOD10 (Medtr3g415590) and ENOD18 (Medtr7g065770), early nodulin-like protein PCY68 (Medtr2g090575), the bidirectional sugar transporter SWEET11 (Medtr3g098930), defensin related PDF44 (Medtr8g010280), nodule-specific PLAT domain protein NPD1 (Medtr2g103303), and eleven Nodule-specific Cysteine-Rich (NCR) genes in response to rhizobia. These genes play essential roles in symbiosis formation, nodule organogenesis, and autoregulation of nodulation (Durgo et al., 2015; Kryvoruchko et al., 2016; Trujillo et al., 2019; Burghardt et al., 2020; Roy et al., 2020).

The R1 trajectory set (four genes) was highly induced at 48 hpi in rhizobia-inoculated roots. This cluster includes genes encoding the CLAVATA3 (CLV)/EMBRYO SURROUNDING REGION (ESR)-RELATED 12 CLE12 peptide (Medtr4g079630), and the transmembrane protein EPFL19 (Medtr8g090010). Trajectory set R6 identified transcripts for the early nodulin protein ENOD11 (Medtr3g415670) that was induced at 12 hpi in rhizobia-inoculated roots. Three trajectory sets (R3, R4, R5) displayed a transient up and then down pattern in rhizobia-inoculated roots compared with continuous non-expression in the control. Trajectory set R3 (five genes) displayed a transient upregulation at 48 hpi and includes transcripts for a UDP-glucosyltransferase family protein (Medtr0036s0220), a frigida-LIKE protein (Medtr7g056317), and a transmembrane protein (Medtr7g100130). Trajectory set R4 (five genes) had an expression peak at 24 hpi in rhizobia-inoculated roots. This trajectory set included transcripts for Nod factor receptor 5 LYR1 (Medtr8g078300), and a peptide transporter PTR3-A-like protein (Medtr7g498330). Trajectory set R5 (two genes) identified an upregulation pattern at 12 hpi in rhizobia-inoculated roots and contained transcripts for the hypothetical protein Medtr2g100690, and an ATP-dependent RNA helicase DDX11-like protein (Medtr0147s0050).

In addition to the upregulated trajectory gene sets identified in rhizobia-inoculated roots, GeneShift also detected three trajectory sets that contained downregulated genes in rhizobia-inoculated roots (R7, R10, and R11). Trajectory set R7 (two genes) identified two transcripts that were upregulated at 72 hpi in uninoculated controls roots: cytochrome P450 family 78 protein (Medtr1g097220) and a phenazine biosynthesis PhzC/PhzF family protein (Medtr1g062530). Trajectory R10 (one gene) displayed a transient up and down at 48 hpi in uninoculated control roots. This gene encodes an F-box Leucine-Rich Repeat (LRR) protein (Medtr7g075970). Also, Trajectory R11 (one gene) contained defensin MtDef4.3 (Medtr8g070780) which showed a transient up and down at 12 hpi in the uninoculated control roots. There are only four genes that did not change expression patterns in the rhizobia-inoculated roots. Two genes belong to trajectory set R9 that started from relatively higher expression at 0 hpi and had off expression for the rest of measured time points in both control and rhizobia-inoculated roots. The other two genes belong to trajectory set R12 and had a continuous upward trend in expression from 0 to 72 hpi in both control and rhizobia-inoculated roots.

To understand the effect of rhizobial inoculation in the shoot, GeneShift compared pattern changes over time between control and rhizobia-inoculated in the shoot. Eighty-three percent of shoot genes did not change expression patterns upon rhizobial inoculation. The largest shoot trajectory set S4 (94 genes) showed a first down then up expression pattern at 12 hpi in both rhizobia-inoculated and control shoots. S4 captured the expression of circadian clock transcriptional factor LATE ELONGATED HYPOCOTYL LHY (Medtr7g118330) and circadian clock gene CCA1/LHY/REVEILLE RVE7 (Medtr6g477860). S4 also contained transcripts for six transmembrane proteins, seven light-harvesting complexes, a root phototropism-like protein (Medtr3g062540), and other developmental related genes. Trajectory set S10 (five genes) also possessed first down then up patterns in both conditions, but S10 started at a much higher expression level at 0 hpi compared with S4. S10 includes genes for the staygreen protein Medtr3g088795, growth-regulating factor Medtr5g027030, and aminopeptidase Medtr6g033240. Trajectory set S15 (45 genes) displayed the first up then down expression at 12 hpi. S15 included transcripts for several core clock components: DNA-binding transcription factor TOC1(Medtr4g108880), LUX ARRYTHMO (Medtr4g064730), EARLY FLOWERING ELF4 (Medtr3g070490). S15 also contained cytochrome P450s (three genes), and LRR receptor-like kinases (three genes). Trajectory set S8 (12 genes) showed similar patterns, but the expression level at 0 hpi was higher including genes for the lipid transfer protein nsLTP24 (Medtr4g028360), NADH dehydrogenases (2 genes), and a cytochromes P450 (Medtr3g076530).

Besides non-shifting shoot genes, GeneShift discovered 13 trajectory sets (31 genes) exhibiting a different expression pattern when plants were inoculated with rhizobia. Trajectory set S0 (7 genes) trended up at 72 hpi responding to rhizobia-inoculation and includes genes for auxin response factor 14 (Medtr8g446900), transmembrane proteins (Medtr4g059310 and Medtr2g040680), and a germin family protein (Medtr2g019250). At 72 hpi, trajectory set S9 (two genes) displayed an upregulated pattern in control shoot and a downregulated pattern upon rhizobia treatment. This trajectory set includes genes for auxin-binding protein ABP19a (Medtr2g044040) and hypothetical protein Medtr8g098925. Trajectory set S13 (five genes) exhibited a 12 hpi peak pattern in the control condition and off expression in the rhizobia-inoculated condition including genes encoding GDSL-like lipase/acylhydrolase (Medtr5g022640), chitinase (Medtr1g099350), and hypothetical protein Medtr3g070480. Finally, trajectory set S17 (two genes) showed a similar pattern except they peaked at 24 hpi in the control condition. S17 includes the genes encoding a DUF247 domain protein (Medtr7g059475) and a white-brown-complex ABC transporter family protein (Medtr1g063920).

To interpret the transcriptional dynamics detected by GeneShift, we performed functional enrichment on each trajectory gene set (Figure 4B and Supplementary Table 5). Two root trajectory sets and five shoot trajectory sets compiled by GeneShift displayed functional enrichment (shown in Figure 4A and Supplementary Table 2) (Benjamini-Hochberg corrected *p* <0.001). Trajectory set R0 was enriched for late nodulin protein, peptide transporter (POT family and solute carrier family 15), and metal ion binding, all of which are characteristic of nodulation. Another root trajectory set R1 was enriched for sulfotransferase activity. In the shoot, non-shift 12 hpi down and up trajectory sets S4 and S10 were enriched for regulation of photosynthesis, B-box zinc finger, and Na+ symporter, while 12 hpi up and down trajectory sets S8 and S15 were enriched for oxygen-evolving enhancer protein 3, tyrosine-specific transport protein, different phosphorylase activities, and the CCT motif which often involved in light signal transduction. The shoot shifting trajectory set S13 was enriched for chitinase.

**Figure 5:**
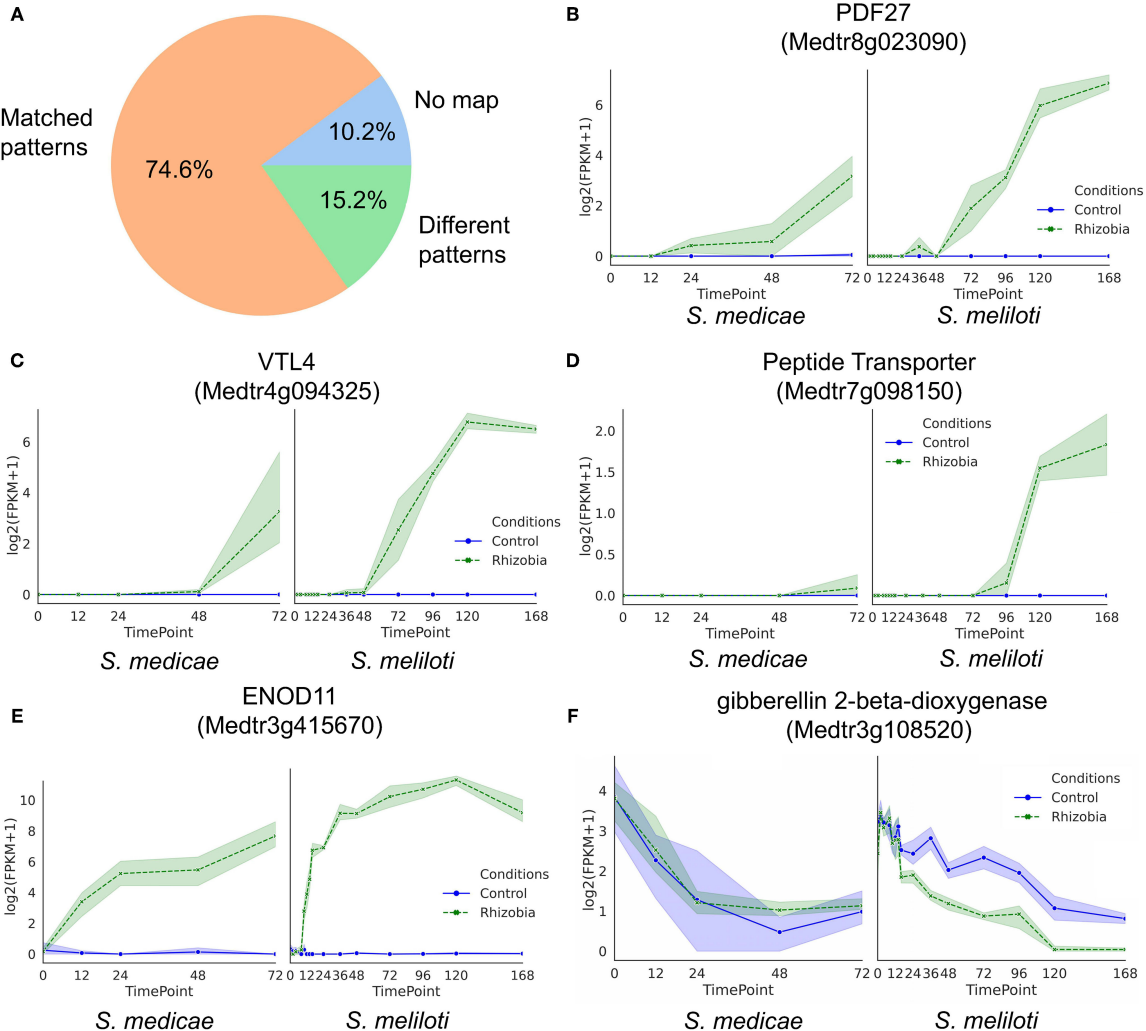
Validation of root time-series expression profiles detected by GeneShift **(A)** Pie chart of GeneShift detected 118 time-series root DEGs expression profiles from our *Sinorhizobium medicae* inoculation *M.truncatula* experiment in comparison to previously published *S. meliloti* induced *M.truncatula* transcriptomic data. **(B–F)** Expression profiles of representative genes in our study *S. medicae* vs. Schiessl study *S.meliloti*. Y-axis, log2 (FPKM+1); x-axis, time (h) after rhizobia inoculation, each dot represents mean value of replicates, and shading indicates confidence interval.

Finally, we asked if the GeneShift-detected gene expression patterns could be validated by previously published RNA-Seq data with a similar experimental system. In Schiessl et al. (2019) *Sinorhizobium meliloti* (*S. meliloti*) was used to inoculate *M. truncatula* jemalong cultivar Jester and 2–3 mm root tissues were harvested at 10 time points (0,2,4,8,10,12,14,16,24,36,48,72,96,120, and 168 hpi). We examined the expression profile of 118 curated GeneShift detected root genes (R2, R8, R9 were excluded and the reason will be explained in the discussion section) in their data set (Figure 5A). The percentage of genes with the same expression pattern over time in both independent experiments was 74.5%. Of the GeneShift discovered root genes, 10.2% were not mapped in the Schiessl et al. transcriptomic study possibly because of their sequence depth and the slight sequence difference between the Jemalong A17 and Jester cultivars. The remaining 15.2% of genes exhibited different expression profiles in their dataset. Figures 5B–F show the expression profiles of representative root genes. Thus, even though experimental differences existed between the two studies, the majority of the GeneShift patterns were corroborated.

## 3. Discussion

A long-standing challenge in any transcriptomic study is to correctly identify DEGs between biologically distinct sample groups (e.g., control vs. treated). Time-series transcriptomic profiling is a powerful approach to understand dynamic biological processes in a window of time as opposed to single start/stop snapshots that might miss co-responsive yet not perfectly synchronized gene regulation. Many time-series transcriptomic studies use well-known software packages to detect DEGs, such as DeSeq2, edgeR, and limma, designed for single time point comparison of aggregated replicates, which is not appropriate for sequential time-series data (Robinson et al., 2010; Love et al., 2014; Ritchie et al., 2015). To address these issues, we developed GeneShift, a workflow that detects similar or distinctive temporal expression patterns between two sample conditions. Although several time-series clustering algorithms are available, these methods can either only handle small GEM input due to implementation constraints (McDowell et al., 2018) or require user input parameters (e.g., k cluster estimates) which can be very challenging to determine (Jung et al., 2017).

GeneShift applies a two-step clustering procedure to ameliorate the input data size problem and three methods to evaluate the two-step clustering performance. The variability of gene expression profiles can also impact the accuracy of DEG identification in time-series studies. Most clustering methods use average values of independently measured expression which might miss useful information (Celeux et al., 2005; Nguyen et al., 2010). GeneShift utilizes each replicate as an independent pattern and sorts the replicates based on their consistency in clustering results. Time point averaging across replicates is unnecessary. GeneShift focuses on the temporal patterns of each replicates, which means small expression changes can be addressed as long as multiple replicates agree with each other. For example, as shown in Figure 5D, peptide transporter Medtr7g098150 exhibited a very small upregulation trend in rhizobia-inoculated roots, validated by another RNA-Seq dataset (Schiessl et al., 2019). GeneShift detects high confidence small expression changes which provide DEGs that might have been overlooked by other approaches.

We applied GeneShift to time-series RNA-Seq data from *M. truncatula* responding to *S. medicae* inoculation and captured 142 root genes and 190 shoot genes with validated patterns in both control and rhizobia inoculated (Table 1). One limitation of GeneShift is it picks up genes with expression differences at 0 hpi which has little biological meaning and is often noise. For example, as shown in Figure 3, the pattern differences across two conditions are driven by 0 hpi dispersed expressions in the R2, R8, R9 trajectory sets. We excluded those 24 genes from further discussion. There were 114 root genes that shifted expression patterns upon rhizobia inoculation. The number of time-series DEGs GeneShift detected was small compared to the previously reported 3,290 single time point DEGs that Schiessl et al. examined using DESeq2 with the threshold of the absolute fold change of over 1.5 and a false discovery rate (FDR) adjusted *p* <0.05. This might be due to the stringency of the 3/3 replicate sorting consistency criterion in our study since GeneShift identified 1,278 root time-series DEGs using 2/3 replicate sorting criteria as depicted in Supplementary Table 6.

There were 62 time-series DEGs that GeneShift discovered that have not been identified by a previous approach over the 12–72 hpi time window (Schiessl et al., 2019). One explanation is that many genes were not mapped in *M. truncatula* cultivar Jester Some of those genes showed a common feature in that the three replicates exhibited the same trajectory pattern, but the three replicate expression values exhibited high variance across the time series. Previous DESeq2 approaches were not able to detect these DEGs because they were categorized as dispersion outliers. One of those genes, Medtr2g089070, a target of transcription factor ABF3, was found in the R0 trajectory set by GeneShift, suggesting GeneShift can identify time-series DEGs based on the full expression pattern. A gene-wise dispersion estimation is implanted in many DEG detection packages like DESeq2 and limma Love et al. (2014); Ritchie et al. (2015), but GeneShift addresses this issue *via* bypassing the need for dispersion estimation.

Most (82.5%) of 114 root time-series DEGs that GeneShift identified were induced around 48 or 72 hpi. Why is this? One possibility is statistical in that the gene expression at early time points fluctuated among replicates making them hard to make into the 3/3 replicate sorting filter we applied. With 2/3 replicate sorting criteria, GeneShift detected 59 early rhizobial induced genes distributed in 24 trajectory sets. The other explanation is biological; the infection threads reach the primordia and nodules are formed around 48 hpi in this system, suggesting a majority of rhizobial triggered transcriptional events happen after 48 h. In addition, the cell-type complexity in the root section we harvested may have diluted some rhizobial related gene expression changes in early infection stages.

Figure 3 summarizes the expression profiles of trajectory sets discovered by GeneShift. GeneShift detected three leginsulin peptide related MtN11/16/17 genes (Legin38: Medtr0416s0030, Legin42: Medtr0093s0090, legin47: Medtr0112s0040) in trajectory set R0. They displayed upregulation in 72 hpi with rhizobia inoculation in the root. Those genes exhibited consistent expression patterns in the Schiessl et al. RNA-Seq dataset as well, suggesting those legin genes are involved in the *M. truncatula* nodulation process regardless of rhizobial partner. Interestingly, a previous study found that Asian soybean cultivars accumulated drastically higher leginsulin than North American soybean cultivars (Krishnan et al., 2015). However, very limited biological functions have been discovered for this hormone-like cysteine-rich peptide.

GeneShift also discovered three defensin related genes that displayed two different expression patterns upon rhizobia inoculation. Both PDF27 (Medtr8g023090) and PDF44 (Medtr8g010280) start trending up from 24 hpi in the rhizobia-inoculated root, whereas another defensin related gene MtDef4.3 (Medtr8g070780) was not induced by rhizobia. MtDef4.3 had an up and down expression pattern at 12 hpi without rhizobia. Phylogenetically, MtDef4.3 and MtDef4.4 are close to a *Prunus persica* defensin gene, which displays antimicrobial activity through specific lipid binding and membrane permeabilization (Kaur et al., 2012; Nanni et al., 2013). This suggests that MtDef4.3 might be involved in controlling the access of rhizobia infection by rhizobia. Besides MtDef4.3, three other genes were also downregulated by rhizobia inoculation (8 genes belonging to the R8 trajectory set were excluded) in roots. These genes encode F-box/LRR protein Medtr7g075970, cytochrome P450 family 78 protein Medtr1g097220, and phenazine biosynthesis PhzC/PhzF family protein Medtr1g062530. As shown in Figure 4, Trajectory set S13 (five genes) was enriched for chitinase, an inducible enzyme group that has been associated with plant defense systems (Boller, 1987). A previous study found chitinase regulates Nod factor levels and infection thread in *Lotus japonicus* (Malolepszy et al., 2018). Our results showed chitinase encoding gene Medtr1g099350 was downregulated by rhizobia in the shoot, suggesting root-shoot signaling involves in the rhizobial induced chitinase pathway. To our knowledge, few genes have been discovered that exhibit time-series downregulation in rhizobia-inoculated plants. Those rhizobial induced down-regulating genes are worthy of further empirical validation.

GeneShift identified 10 different root transporter genes across several trajectory sets. Four peptide transporter genes (Medtr1g026750, Medtr7g098150, Medtr7g098090, and Medtr3g069420) were categorized in R0 and displayed 48 hpi to 72 hpi upregulation in rhizobia-inoculated roots whereas peptide transporter PTR3-A-like protein Medtr7g498330 showed 24 hpi up-and-down expression in rhizobia-inoculated roots. In Arabidopsis, NPF/PTR family proteins transport large substrates and plant hormones (Tsay et al., 1993). Our study suggests peptide transporters are induced by rhizobia at different times depending on the substance they transport. Besides peptide transporters, GeneShift also detected MtSWEET11 (Medtr3g098930), a nodule-specific sugar efflux transporter in trajectory set R0. MtSWEET11 is associated with infection thread and symbiosome membranes of infected cells (Kryvoruchko et al., 2016). A previous study demonstrated the expression of MtSWEET11 increased by 4 dpi (Kryvoruchko et al., 2016), and our study suggests the upregulated expression might start from 12 hpi. As shown in Figure 5C, GeneShift also detected VTL4 (Medtr4g094325), a vacuolar iron transporter in trajectory set R0 that upregulated in rhizobia-inoculated roots at 48 hpi. Iron is a key element during nodule development because iron functions as a co-factor of many metalloenzymes (González-Guerrero et al., 2014). Previous transcriptomic data has demonstrated that VTL4 was explicitly expressed in nodules vs. rhizobia-inoculated roots (Roux et al., 2014; Walton et al., 2020). Besides VTL4, GeneShift also detected an iron man family member, IMA10 (Medtr4g026390), in trajectory set R0. IMA10 also started trending up in rhizobia-inoculated root from 48 hpi. The Iron man family consists of peptides that control iron transportation in plants (Grillet et al., 2018). A previous study has found LjSEN1, a homolog to VTL1 in *Arabidopsis thaliana* was expressed exclusively in nodule infected cells and continuously increased from 10 to 24 dpi (days post inoculation) in *L. japonicus* (Hakoyama et al., 2012). They also found LjSEN1 is essential for bacteroid differentiation in nodule development. Our findings confirm the crucial role of iron in nodule development and refine the time of induction of those gene expressions by rhizobia. Medtr4g094352, MtN21/EamA nodulin-like protein is one of the transporter encoding genes classified into trajectory set R0 by GeneShift. A previous study demonstrated an enhanced vascular immunity in an AtUMAMIT5 (UMAMIT is the name of MtN21/EamA-like transporter family in *A. thaliana*) mutant suggesting it might modify sap composition and acts as an inhibitor for xylem-infected pathogens (Denancé et al., 2013), but this needs to be validated with more experiments. The gene for MtABCG9 (Medtr8g059150), an ATP-binding cassette (ABC) transporter also induced by rhizobia in root from 48 hpi. A recent study showed the expression of MtABCG56, another gene in the ABC transporter family, is induced by rhizobia from 6 hpi and it mediates cytokinin transportation which is crucial for nodule development (Jarzyniak et al., 2021).

GeneShift discovered three early nodulin genes which were induced by rhizobia treatment at different times. ENOD10 (Medtr3g415590) and ENOD18 (Medtr7g065770) were both categorized in trajectory set R0 with a 72 hpi upregulation pattern in rhizobia-induced roots. However, ENOD11 (Medtr3g415670), encoding a putative cell wall repetitive proline-rich protein, was categorized in trajectory set R6 with a 12 hpi upregulation pattern in rhizobia-induced roots. ENOD11 has been used as an early infection marker gene, which is consistent with the expression pattern detected by our study (Journet et al., 2001). The range of times of rhizobial induction suggests more roles for ENOD genes are involved during nodule development. GeneShift also detected Plantcyanin PCY70 (Medtr4g130780) and PCY68 (Medtr2g090575), which are two early nodulin-like genes in trajectory set R0. AtENODL14 (At2G25060), a homolog of PCY70, was identified in a phosphoproteomic analysis of plasma membranes treated with effectors of plant immunity (Benschop et al., 2007; Denancé et al., 2014). More experimental evidence is required to elucidate the functions of ENOD and ENODL genes.

GeneShift validated the rhythmic expression pattern of several key players in circadian clock control such as TOC1 (Medtr4g108880), LHY (Medtr7g118330), ELF4 (Medtr3g070490), LUX (Medtr4g064730), and RVE7 (Medtr6g477860). Our results are consistent with the previous report on the transcriptional behaviors of TOC1 and LUX in the leaves of 28 day *M. truncatula* ecotype R108 inoculated with *S. meliloti* (Kong et al., 2020). Their study also suggests that LUX is involved in nodule development because of the reduced nodule number in two Tnt1 insertion mutants, although more experimental study needs to be done to support the statement. The recent study also revealed the expression pattern we observed with those clock-associated transcripts is also displayed in the root and nodule (Achom et al., 2021).

In conclusion, legumes utilize a long-distance signaling pathway to regulate nodulation. The nodule development process is accompanied by massive transcriptional reprogramming including the activation and repression of sets of genes. We present transcriptomic data on plants responding to rhizobia from both root and shoot tissue and a computational workflow which will serve as a useful resource for future nodulation studies. The GeneShift workflow provides a powerful way to profile the time-course transcriptional response to rhizobia during nodulation. We note that the GeneShift workflow can also be applied to any time-series transcriptomic datasets and is a general purpose option for time series transcriptome analytics.

## 4. Materials and Methods

### 4.1. Biological Material Harvest and Rhizobial Inoculation

*Medicago truncatula* A17 plants were grown, inoculated and root sections harvested as in Poehlman et al. (2019). Specifically, *M.truncatula* A17 seeds were scarified for 5–8 min in sulfuric acid, rinsed 5 times with distilled water, washed with 3% bleach, rinsed another 5 times in distilled water, and imbibed in water with gentle rocking for 2 h at room temperature. Seeds were then placed in a moist chamber (petri dish) at 4°C for 48 h in the dark, followed by germination at room temperature for 24 h in the dark. The germinated seedlings (radicals about 1–2 cm) were placed on an aeroponic chamber in nodulation media as described previously (Penmetsa and Cook, 1997) under a 16h/8h light/dark cycle. At 3.5 h into the light cycle on the third day after loading onto the apparatus, a set of plants was marked with ink 1 cm from the root tip (at the distal end of the rhizobia-susceptible root maturation zone) to be used for tracking the location of the nodule susceptibility zone and 2 cm root sections starting 1 cm from the root tip were harvested from 10 experimental plants (0h sample). *S. medicae* ABS7 (150 OD600 units) in nodulation medium or bacteria-free nodulation medium (mock inoculation) was then added to the apparatus. Tissue sections 2 cm long were harvested from the nodule susceptibility zone from 10 plants each at 12, 24, 48, and 72 hpi, using the marked plants to determine the location of the developing nodules. For shoot samples, we harvested all tissue above the hypocotyl from the same plants. Three biological replicates of the time course for both inoculated and uninoculated samples were collected for RNA-Seq analysis.

### 4.2. RNA Isolation, Library Preparation, and Sequence Data Processing

RNA was isolated from *M. truncatula* root and shoot samples using the E.Z.N.A.Â® Total RNA Kit (Omega Bio-tek, USA) according to the manufacturer's protocols. RNA libraries were made and sequenced by Novogene Co., 740 Ltd. (Beijing) from 100 to 1,000 ng of total RNA prepared by a stranded kit (Illumina TruSeq Stranded 741 Total RNA Kit or NEB Next UltraTM II Directional RNA Library Prep Kit for Illumina). These libraries were included in a final dataset consisting of 60 libraries, including 30 libraries from this study (three replicates of five time points each for inoculated and uninoculated wild type (A17) shoot segments) and 30 libraries previously reported from inoculated and uninoculated wild type (A17) root segments (Poehlman et al., 2019). The PBS-GEM workflow (https://github.com/wpoehlm/PBS-GEM) was used to process RNA sequencing reads on Clemson University's Palmetto Cluster (Pertea et al., 2016). Poor quality reads and adapters were removed using Trimmomatic-0.38 (Bolger et al., 2014). Next, cleaned reads were mapped to the Mt4.0v1 reference genome using hisat2-2.1.0 (Kim et al., 2015). Gene and transcript abundances were estimated using stringtie-1.3.4d (Pertea et al., 2015). We also processed 134 RNA-Seq samples from a previous study (Schiessl et al., 2019) using GEMmaker (https://github.com/SystemsGenetics/GEMmaker).

### 4.3. Time-Course Expression Clustering Using GeneShift Workflow

GeneShift is a workflow that enables the detection of gene expression pattern changes over time from two different conditions as shown in Figure 2. GeneShift begins with a time-series gene expression matrix where it produces a set of gene cluster shift status accompanying biological replicate sorting and plots showing different expression patterns between the two conditions. The GeneShift workflow takes advantage of soft-DTW-KMeans (Cuturi and Blondel, 2017) and DP_GP (McDowell et al., 2018) to perform a high quality clustering. Below are is the six phases of the GeneShift workflow.

#### 4.3.1. Gene Expression Matrix

The two input datasets for the GeneShift workflow were an *M. truncatula* shoot GEM and a root GEM (Figure 2A). Each GEM combined three replicates and two conditions: inoculated and uninoculated. Quantile normalization and log2 transformation were applied to ensure heteroskedasticity and suitable comparison between different time points as depicted in Supplementary Figure 1. Before GeneShift clustering of the gene expression patterns, we extracted an “off” GEM consisting of all unexpressed genes which did not need to be clustered. Unexpressed genes were determined by expression sorting. If one biological replicate was 0.00 fragments per kilobase of gene per million read pairs (FPKM) through all the measured time points, the gene expression under that condition was categorized as unexpressed.

#### 4.3.2. Initial Clustering

GeneShift applied soft-Dynamic Time Warping (DTW) K-means clustering to perform this unsupervised learning task (Cuturi and Blondel, 2017). K-means is one of the fastest clustering algorithms that separate samples in k groups and minimizes the within-cluster sum-of-squares criterion. We used DTW score with K-means clustering instead of the default Euclidean distance, to compute the best possible alignment for comparing time series. Soft-DTW is a differential loss function that can compute the soft-minimum of all costs by all possible alignments between two time series (Cuturi and Blondel, 2017). We utilized the python tslearn package to perform the initial clustering (Tavenard et al., 2020). For each treatment, all *M. truncatula* expression patterns are separated into k groups as depicted in Figure 2A, and we iterated through *K*-values ranging from 35 to 90 with a step size of 5.

#### 4.3.3. Fine Clustering

This step captures the temporal shifts in the initial clustering outputs (shown in Figure 2C). We used the DP_GP_cluster to fine cluster the expressions in each k group. DP_GP_cluster is a software that models gene expression trajectory using a Dirichlet process Gaussian process model (McDowell et al., 2018). We ran DP_GP in fast mode with specified sample iteration parameter: max_num_iterations = 1,000.

#### 4.3.4. Choose Optimal Clusters

We performed three analyses to choose an optimal value for n_clusters. We selected k ranging from 35 to 90 as described in initial clustering and fine clustering each k cluster output *via* DP_GP_cluster. We next calculated the Davies-Bouldin index (Davies and Bouldin, 1979) Calinski-Harabasz index (Caliński and Harabasz, 1974), and silhouette coefficient (Rousseeuw, 1987) for each k depicted in Supplementary Figures 2A,B. In GeneShift, the performance at each k was calculated using the python scikit-learn package: 'sklearn.metrics.davies_bouldin_score'; 'sklearn.metrics.calinski_harabasz_score'; 'sklearn.metrics.silhouettescore'. When the Davies-Bouldin index is closer to zero, it indicates a better partition. When the Calinski-Harabasz index is higher, clusters are dense and well separated. The Silhouette score is bound between –1 for incorrect clustering and 1 for highly dense clustering, and scores around zero indicate overlapping clusters. As shown in Supplementary Figure 2, the value of 50 and 55 are the best picks for the given *M. truncatula* root data and *M. truncatula* shoot data based on their performances in three tests.

#### 4.3.5. Deep Learning Classification Model

To evaluate the ability of GeneShift to identify time-series expression patterns from GEM, we used neural network classification. Briefly, we used an RNN-LSTM as a training model with a 30 train test split of data to evaluate GeneShift classification performance. Each trajectory set had to contain at least two samples or else the confusion matrix will not be aligned due to the train test split. The details of the LSTM model can be retrieved from the GeneShift github repository. The output is a confusion matrix as shown in Supplementary Figure 3 and the F1 score for the shoot data is 83% and for the root data is 84%.

#### 4.3.6. Post-clustering Analysis

After selecting the optimal number of pattern clusters, we used replicate sorting to obtain high quality expression shifting status between control and treated conditions. If two or three replicates of the one gene expression are categorized into the same trajectory cluster, it was qualified for the comparison between conditions. For three out of three replicate sorting, we identified 142 qualified genes falling into 14 expression patterns in *M. truncatula* root and 190 qualified genes falling into 19 expression patterns in *M. truncatula* shoot depicted in Table 1. In the *M. truncatula* root, 138 genes shift expression trajectory from control to rhizobia condition and 4 genes remain the same expression trajectory under two conditions. In *M. truncatula* shoot, only 31 genes exhibited a shift in expression trajectory between the two conditions, and the rest of 159 genes showed the same expression trajectory under both conditions. Supplementary Table 6 depicts the genes where 2/3 replicates are sorted in *M. truncatula* root and shoot. We next further categorized shifted genes based on their expression patterns under two conditions as depicted in Figure 2F and Table 1.

To interpret shifting and non-shifting expression patterns, we performed functional enrichment to the genes assigned to each GeneShift index using FUNC-E (https://github.com/SystemsGenetics/FUNC-E). Enriched terms were identified using Fisher's exact test with a significance threshold of p <0.001 after Benjamini and Hochberg correction (Benjamini and Hochberg, 1995). Gene model annotations for the Mt4.0v1 genome were obtained from Phytozome (Goodstein et al., 2012) and parsed as input.

## Data Availability Statement

The datasets presented in this study can be found in online repositories. The names of the repository/repositories and accession number(s) can be found below: National Center for Biotechnology Information (NCBI) BioProject database under accession number PRJNA554677. The GeneShift source code is available at https://github.com/yueyaog/GeneShift. The root and shoot FPKM GEMs used in this analysis are freely available at https://figshare.com/s/32244d053a8c987b639e.

## Author Contributions

YG, JF, and FF designed the research. YG and BS developed the software. ES and SC harvested and prepared RNA libraries. ES and WP processed the RNA-Seq data. YG analyzed data. YG and FF wrote the manuscript. All authors contributed to the article and approved the submitted version.

## Funding

This study was supported by the National Science Foundation award IOS 1444461 to JF and FF.

## Conflict of Interest

WP was employed by Sage Bionetworks. The remaining authors declare that the research was conducted in the absence of any commercial or financial relationships that could be construed as a potential conflict of interest.

## Publisher's Note

All claims expressed in this article are solely those of the authors and do not necessarily represent those of their affiliated organizations, or those of the publisher, the editors and the reviewers. Any product that may be evaluated in this article, or claim that may be made by its manufacturer, is not guaranteed or endorsed by the publisher.
