# Supplementary material for: Time Series Transcriptome Analysis in Medicago truncatula Shoot and Root Tissue During Early Nodulation
Source: Front Plant Sci. 2022 Apr 7;13:861639. doi: 10.3389/fpls.2022.861639 (PMC9021838; doi:10.3389/fpls.2022.861639)
Supplement: Supplementary file 2 [file Image_1.pdf]

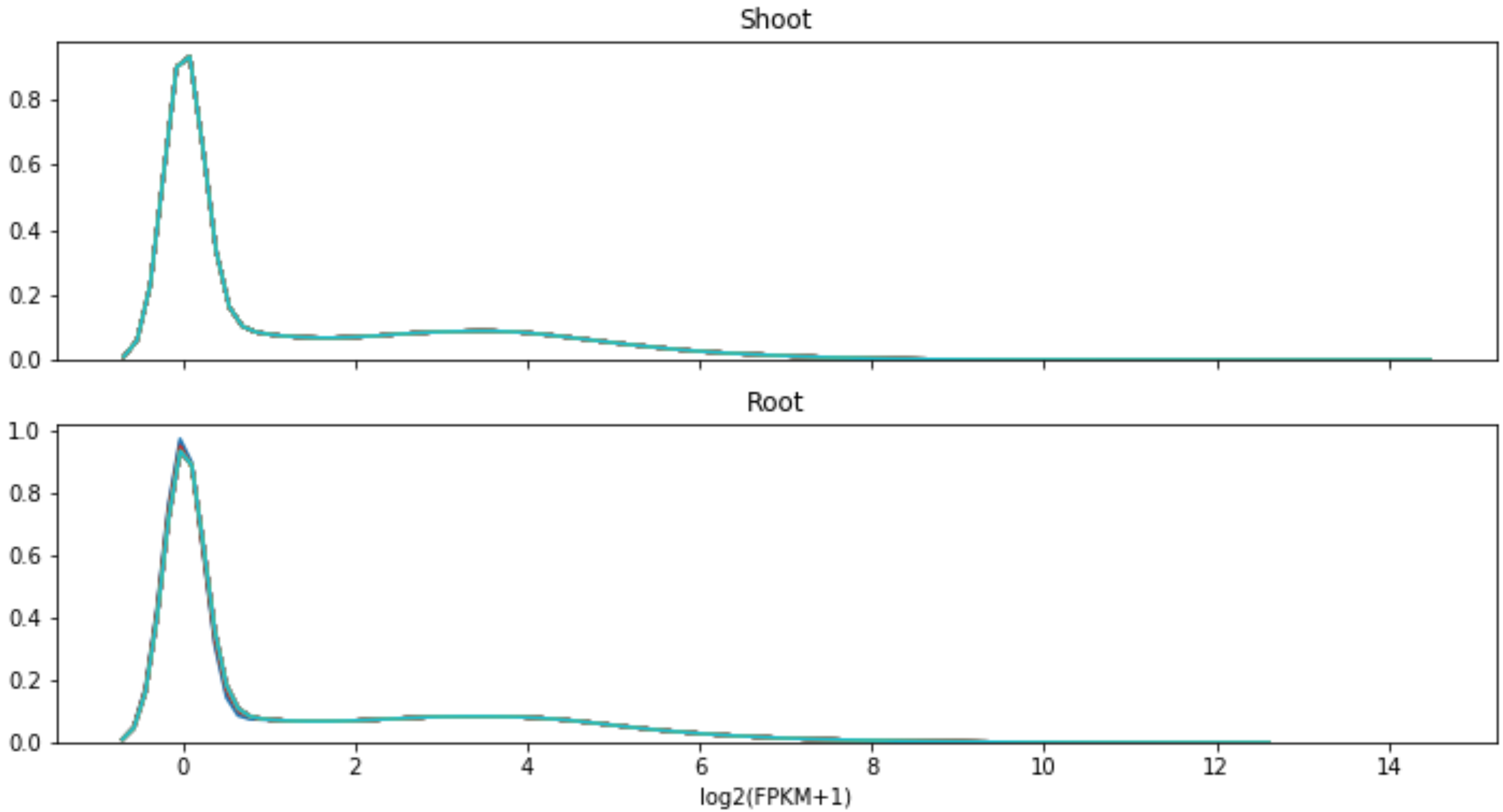

Supplemental Figure 1. Sample distribution for *Medicago truncatula* root and shoot gene expression matrices.
