# Supplementary material for: Time Series Transcriptome Analysis in Medicago truncatula Shoot and Root Tissue During Early Nodulation
Source: Front Plant Sci. 2022 Apr 7;13:861639. doi: 10.3389/fpls.2022.861639 (PMC9021838; doi:10.3389/fpls.2022.861639)
Supplement: Supplementary file 3 [file Image_2.pdf]

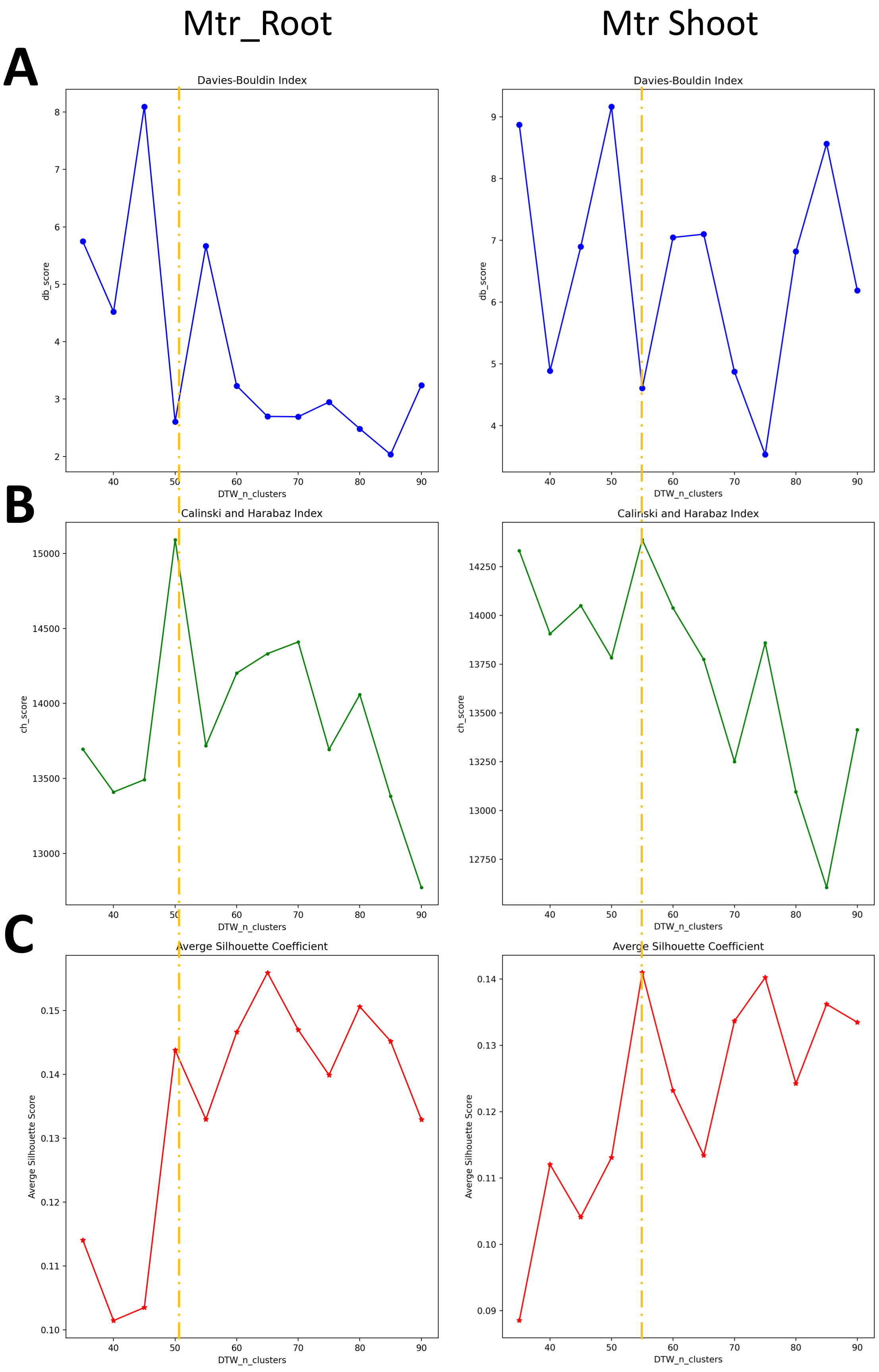

Supplemental Figure 2. Estimate of the Number of GeneShift Clusters for *M. truncatula* Root and Shoot Data. Three methods were used to estimate the number of GeneShift clusters. A. Davies-Bouldin index measured similarity between clusters. DB index values were measured for the number of DTW clusters ranging from 35 to 90 with the step size of 5. B. Calinski and Harabaz index measured separation and density of each cluster. CH indexes were measured for the number of DTW clusters ranging from 35 to 90 with the step size of 5. C. Silhouette coefficient relates to clusters quality. Average silhouette coefficients were measured for the number of DTW clusters ranging from 35 to 90 with the step size of 5. Vertical yellow lines represent the optimal number of clusters for root and shoot.
