# Supplementary material for: Time Series Transcriptome Analysis in Medicago truncatula Shoot and Root Tissue During Early Nodulation
Source: Front Plant Sci. 2022 Apr 7;13:861639. doi: 10.3389/fpls.2022.861639 (PMC9021838; doi:10.3389/fpls.2022.861639)
Supplement: Supplementary file 4 [file Image_3.pdf]

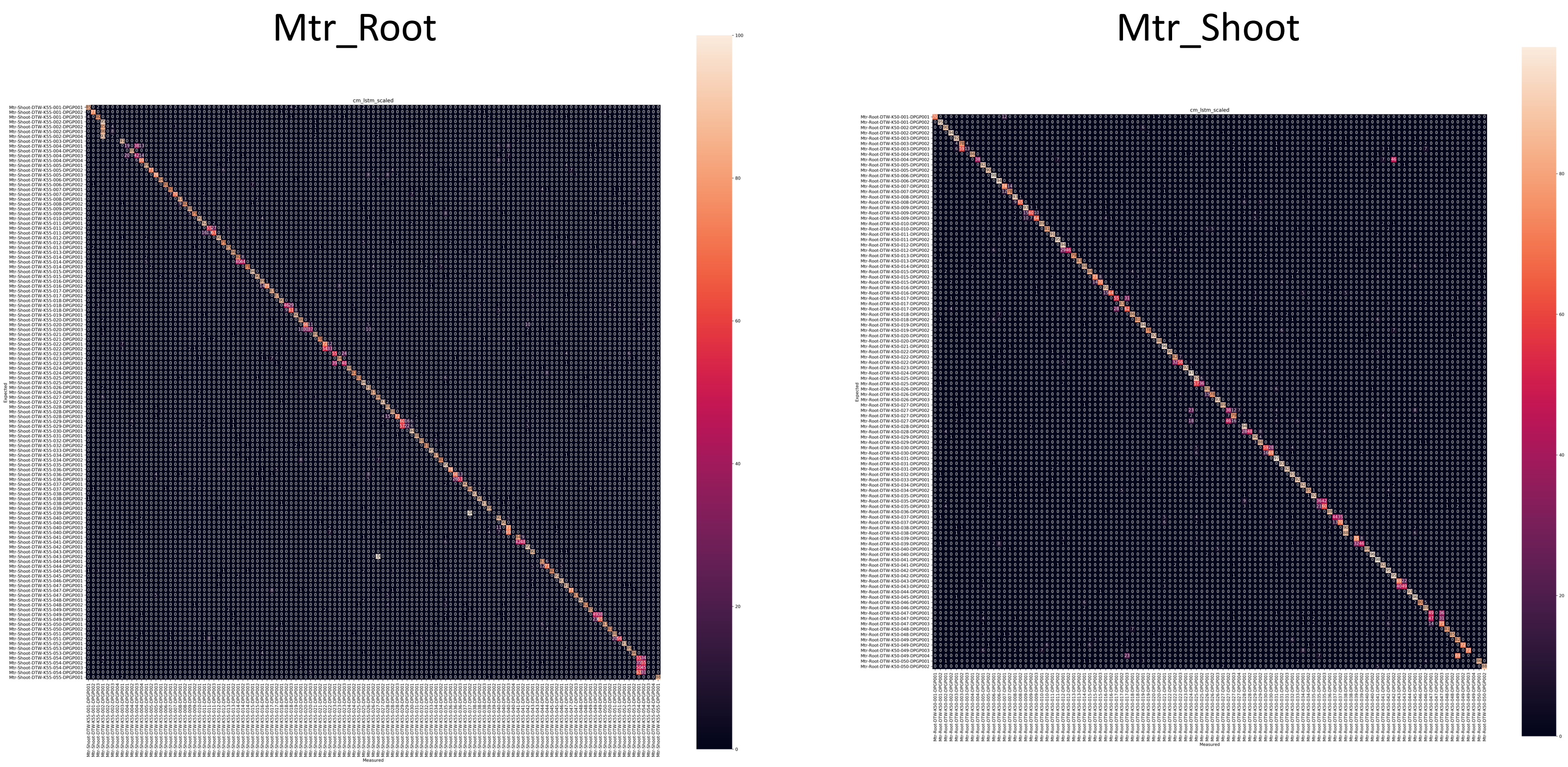

Supplemental Figure 3. Evaluation of Classification Quality of GeneShift on the *M. truncatula* Root and Shoot Data Sets**.** A confusion matrix was used to evaluate GeneShift classification performance. The x axis represents the labels predicted RNN LSTM model. The y axis represents the true labels generated by GeneShift workflow. The diagonal elements represents the number of expression which the predicted label matches true label.
